# Study on a risk model for prediction and avoidance of unmanned environmental hazard

**DOI:** 10.1038/s41598-022-14021-3

**Published:** 2022-06-17

**Authors:** Chengqun Qiu, Shuai Zhang, Jie Ji, Yuan Zhong, Hui Zhang, Shiqiang Zhao, Mingyu Meng

**Affiliations:** 1 Jiangsu Province Intelligent Optoelectronic Devices and Measurement-Control Engineering Research Center, Yancheng Teachers University, Yancheng, 224007 China; 2 School of Automotive and Traffic Engineering, Jiangsu University, Zhenjiang, 212013 China; 3 Anhui Institute of Optics and Fine Mechanics, Chinese Academy of Sciences, Hefei, 230031 China; 4 Interdisciplinary Graduate School of Science & Engineering, Tokyo Institute of Technology, Yokohama, 2268502 Japan

**Keywords:** Environmental sciences, Environmental social sciences

## Abstract

Comprehensive research is conducted on the design and control of the unmanned systems for electric vehicles. The environmental risk prediction and avoidance system is divided into the prediction part and the avoidance part. The prediction part is divided into environmental perception, environmental risk assessment, and risk prediction. In the avoidance part, according to the risk prediction results, a conservative driving strategy based on speed limit is adopted. Additionally, the core function is achieved through the target detection technology based on deep learning algorithm and the data conclusion based on deep learning method. Moreover, the location of bounding box is further optimized to improve the accuracy of SSD target detection method based on solving the problem of imbalanced sample categories. Software such as MATLAB and CarSim are applied in the system. Bleu-1 was 67.1, bleu-2 was 45.1, bleu-3 was 29.9 and bleu-4 was 21.1. Experiments were carried out on the database flickr30k by designing the algorithm. Bleu-1 was 72.3, bleu-2 was 51.8, bleu-3 was 37.1 and bleu-4 was 25.1. From the comparison results of the simulations of unmanned vehicles with or without a system, it can provide effective safety guarantee for unmanned driving.

## Introduction

In the automobile industry, unmanned driving technology has attracted a great deal of attention in recent years. It can fundamentally change the automobile industry and traffic systems. On the other hand, it can also alleviate the problems of accidents, pollution, and congestion of existing vehicles and the traffic^1^.

The commercialization of the unmanned driving should take safety as the premise and realize the importance of the safe unmanned driving in the complex driving environment^2–4^, which is the theme of this paper.

Anti-collision technology is one of the key points of unmanned research. Many achievements have been made in the development of anti-collision technology, such as sensor information fusion, anti-collision research, and anti-collision warning strategy^4–6^. However, it is still a long distance from being completely practical considering the influence of multiple working conditions.

Some scholars have concluded the problems as follows:

Limited information fusion. At present, the research on sensor fusion is only the fusion between two or three kinds of sensors, and the information of fusion cannot cover the working conditions overall^5–7^. In order to adapt to the actual driving conditions, it is necessary to fuse the information of various sensors and other data sources.

Multiple road conditions studies are incomplete. No overall consideration is given to factors such as road environment, weather conditions, the influence of personnel in the environment, and the fastest response speed of vehicles^8–11^.

The early warning strategy needs improving. The present study basically takes distance as the evaluation index. However, for the actual traffic situation, the process from safety to danger is a gradual change, and multiple evaluation indexes should be used^9–13^.

To solve the problems above, this paper adopts the idea of dynamic risk assessment based on the historical data of the environment and predicts the risk by priority based on the results of environmental risk assessment^14–17^. The integration of the booming internet big data industry and electronic information engineering technology makes the risk assessment of traffic environment no longer rely on manual rule setting and machine vision recognition, but can realize joint modeling and statistical analysis by using navigation applications and data from the transportation department 10. Moreover, it is possible to dynamically assess the risk of the environment based on historical circumstances and reapply the assessment results to the risk prediction of specific objectives in the environment^18–20^. Therefore, this train of thought has high practical significance and application value.

Target detection is the leading technology of hazard prediction. The current target detection is mainly aimed at pedestrian, traffic sign, or obstacle^21–23^. In 2019, It proposed an improved SSD_ARC algorithm for key target detection tasks in driving scenarios^24–27^. This method can realize fast multi-objective recognition, semantic annotation, and positioning box selection. Although it provides a general recognition framework, it does not involve the risk of identifying the environment itself. VAR system (TTV system) is to have a portable system which can be used for any type of LCD system and help the referees to count the ball better during the game to have a more qualified game^28^. The AGV (automated guided vehicle) is a system which typically made up of vehicle chassis, embedded controller, motors, drivers, navigation and collision avoidance sensors, communication device and batteries, some of which have load transfer devices^29–33^. By contrast, this system makes up for this omission by adopting the idea of the priority of the big data risk conclusion model and supplement of target detection, which has high practical significance and application value^34–47^. On this basis, this paper proposes an improved SSD method by two steps:

First, the positive sample and negative sample imbalance of SSD are improved by FL loss function.

The second is to improve the common boundary box selection and matching in the target detection algorithm.

This paper presents an environmental hazard prediction and avoidance system. The system is divided into two parts: the first part is the prediction part, which is divided into three levels, including environmental perception, environmental risk model, and target detection. The second part is the avoidance part. According to the results of hazard prediction, conservative driving strategy based on speed limit is adopted. By using this system, vehicles can slow down in high-risk areas or traffic complex environments and increase their speed when the risk is low.

The core of safe driving lies in avoiding danger. However, avoiding danger will inevitably affect driving speed and comfort, especially avoiding environmental danger, which is mainly accomplished by implementing a defensive driving strategy. Therefore, the most important part of this paper is the prediction of whether to use the defensive driving strategy. The prediction section first identifies the environment, such as identifying the intersection, lane, parking lot, and pedestrian crossing near the primary and secondary school campus, and then evaluates the risk and gives the forecast target and priority according to the historical data. Finally, the risk index of the target is predicted separately and evaluated synthetically.

At present, the visual algorithm of environment perception can complete the task of environment recognition^42,48–60^. By combining LBS positioning and other methods, the environmental information can be preset in advance, and the recognition speed and accuracy of the visual algorithm can be improved at the same time. Environmental risk assessment algorithm uses deep learning technology, with the help of Internet open traffic accident database, the comprehensive analysis of traffic accident affected factors in order to rank the dangerous objectives in the environment. It is feasible to practice the idea, but predicting priorities will take a lot of testing to finalize. Besides, under unknown circumstances, the hazard prediction algorithm has been realized in the conflicts of people and cars. The risk prediction is still being explored and the risk error of traffic environment under different time, weather, and other factors need further correcting. The structure of thesis is divided into four aspects:

Section 1: This paper briefly introduces the development of automobile safety and anti-collision technology and explains the importance of anti-collision technology to the driverless. Although the environmental hazard prediction and avoidance system has not been developed, the significance and prospect of this system are expounded.

Section 2: There are four kinds of target detection methods commonly used in unmanned driving. In this design, we will focus on the risk prediction based on deep learning.

Section 3: Hardware and software design of environmental hazard prediction and avoidance system.

Section 4: The main function of the environmental hazard prediction avoidance system is to avoid the risk. With MATLAB, CarSim software for simulation, we can eventually obtain the experimental results to prove the feasibility of the system design implementation.

## System model

The realization of the environment awareness system mainly includes four steps: Firstly, it can input the positioning data to the system with BDS/GPS satellite positioning, LBS positioning of WIFI and base stations. Secondly, it can also use the electronic compass module to achieve position refinement. Moreover, the environment prejudgment’s realizing is based on position and machine vision. Finally, the environmental data is output. Among them, the methods of satellite positioning and electronic compass positioning are quite mature, but how to achieve environmental judgment and corresponding risk assessment on this basis is the key problem to be solved by the environmental awareness system. In this paper, a risk model is established based on location, accident data, and a target detection algorithm through depth learning, which is proposed to realize environmental judgment.

### Risk model based on location and accident data

According to the location information provided by the satellite and the electronic compass, it is possible to make judgments on the types of nearby environment. There are six categories of judgments: residential land, industrial land, public facilities land, commercial building land, transportation facilities land, and road land.

Since most driverless vehicles using this system are running, making detailed perception based on machine vision more necessary, which can be divided into two types: intersection and road. Driving environment types are shown in Table 1. Intersections can be divided into three types: three branches, four branches and, multiple branches. And roads can be divided into four types: expressways, main roads, secondary roads, and branch roads. Allowing for two points above, so as to obtain the judgment of the driving environment.

**Table 1:** This table mainly describes the driving environment classification and driving environment type.

Classification of driving environment	Type of driving environment
Intersections	Three branches
Intersections	Four branches
Intersections	Multiple branches
Roads	Expressway
Roads	Main road
Roads	Secondary trunk road
Roads	Branches

The driving environment classification includes intersection and road. The driving environment type is divided into different branches and roads. Allowing for two points above, so as to obtain the judgment of the driving environment.

Based on the environment-aware data and the type of the nearby environment, the specific name of the nearby environment can be obtained. At the same time, the system can carry out Internet communication and obtain real-time traffic and weather conditions. Allowing for the above information, environmental risk can be judged from the following three aspects. First of all, risk judgment is ultimately to judge risk types and risk objectives. Secondly, they can be summarized as car–car conflict risk, car–person conflict risk, car–object conflict risk, and vehicle control risk. Eventually, risk objectives are visual objectives such as vehicles, pedestrians, bicycles and electric vehicles. Moreover, risk itself is divided into real risk and hidden risk, and real risk is the possibility of collision between risk target and vehicle on site. The hidden risks are difficult to confirm due to various reasons. But there is still the possibility of collision.

#### Location based

Risks based on nearby environment types are shown in Table 2. The types of nearby environment can be divided into several major categories such as residential, industrial, public facilities, commercial and transportation facilities environment. And the traffic facilities environment refers to bus stations, railway stations, airports, subway stations and other passenger transport hubs.

**Table 2:** This table mainly describes the types of accessories, main risk types and main risk objectives.

Type of nearby environment	Main risk types	Major risk objectives
Reside	Car–person conflict	Non-motor vehicle, pedestrians
Industry	Car–car conflict/vehicle control	Vehicle
Public facilities	Car–person conflict	Non-motor vehicle, pedestrians
Commerce	Car–person conflict	Non-motor vehicle, pedestrians
Means of transportation	Car–car conflict/car–person conflict	Vehicle, non-motor vehicle

The nearby types are divided into residence, industrial land, etc., the main risk types are divided into vehicle person conflict, vehicle control, etc., and the main risk objectives are divided into non motor vehicles, pedestrians, etc.

#### Time based

Through the analysis of traffic data on roads, we can sort out the traffic flow of roads at different times. Generally speaking, large traffic volume and complicated traffic environment in each environment represent greater risks, such as working days, holidays and rush hours, could affect the traffic flow. For example, whether there is a road design for non-motor vehicle isolation design and the type of environment in which it is located all affect the complexity of traffic.

For working days, the risk of car–car conflict is more significant in most environments, and the same environment type is different in specific environment. For public facilities, full-time educational facilities have a significant risk of collision between people and vehicles after school. Besides, cultural facilities such as public libraries and museums have a higher risk during holidays, and medical facilities have different situations. For the business environment, different commercial districts have different traffic time distributions.

Therefore, this part of the program needs to input the current time. The specific risk type and priority of risk objectives will be determined through database queries.

#### Based on the scene

Due to possible errors in location and database, the system will confirm and supplement the on-site target detection. First, targets such as pedestrians and vehicles are detected ahead. Compared with the above judgment results, the existing results are marked, or unexpected obstacles appear on site. This part is identified through machine vision to add the non-existent results and avoid the omission of risk targets.

Since the risk targets are divided into real risks and possible risks, the existing risk targets such as vehicles and pedestrians are identified by the completed target detection algorithm, and risk weighting under space–time conditions is evaluated based on historical information.

**Figure 1:**
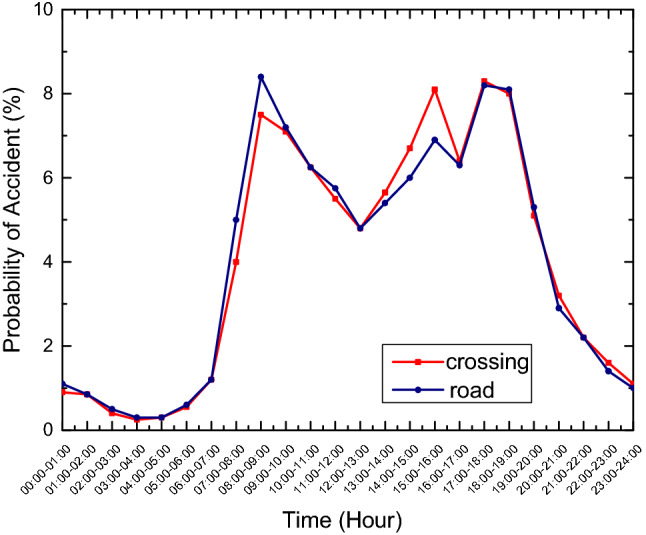
The accident incidence rate in hours of crossing and road in shanghai are fitted by linear relationship. It can be seen from the figure that the value is high from 6 a.m. to 8 p.m.

**Figure 2:**
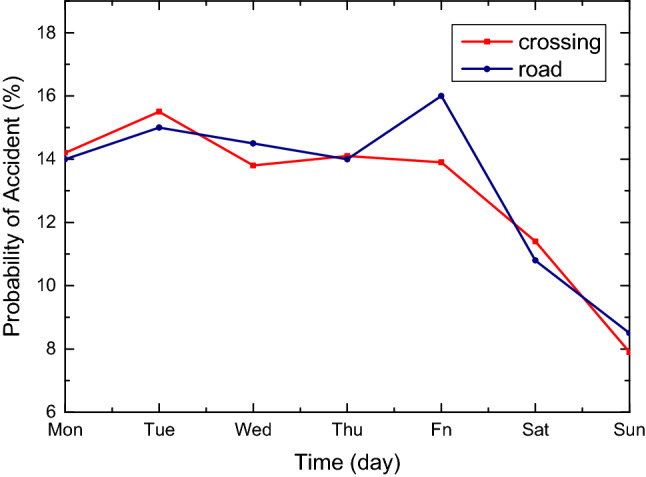
The accident incidence rate in weeks of crossing and road in shanghai are fitted by linear relationship. It can be seen from the figure that the overall trend is downward.

Figures 1 and 2 show the accident rate in hours and a week respectively based on the data from Shanghai. The appropriate model is generated with specific data.

The accident rate varies greatly with time, and vise verse. Weighted value table based on accident ratio is shown in Table 3. With the average value of 1 for each type, the accident data of the whole year are used for statistics, and the weighted value of each time period is re-weighted.

**Table 3:** The table shows the probability of road and cross accidents from 1 a.m. to 6 p.m. from Monday to Sunday.

Day	Time								
	Type	01:00	02:00	08:00	09:00	10:00	11:00	12:00	18:00
Monday	Road	0.22	0.20	1.11	2.05	1.91	1.74	1.47	2.30
	Crossing	0.21	0.19	1.32	2.26	1.91	1.69	1.52	2.17
Tuesday	Road	0.24	0.22	1.21	2.24	2.09	1.90	1.60	2.51
	Crossing	0.28	0.21	1.42	2.42	2.04	1.81	1.63	2.33
Wednesday	Road	0.22	0.20	1.09	2.02	1.89	1.72	1.45	2.27
	Crossing	0.27	0.20	1.36	2.33	1.96	1.74	1.57	2.23
Thursday	Road	0.22	0.21	1.11	2.06	1.93	1.76	1.48	2.31
	Crossing	0.27	0.20	1.34	2.29	1.93	1.71	1.54	2.20
Friday	Road	0.22	0.20	1.08	2.01	1.87	1.71	1.44	2.25
	Crossing	0.29	0.22	1.46	2.50	2.11	1.87	1.68	2.40
Saturday	Road	0.17	0.16	0.87	1.60	1.50	1.37	1.15	1.80
	Crossing	0.20	0.14	0.99	1.68	1.42	1.26	1.13	1.62
Sunday	Road	0.13	0.12	0.63	1.16	1.09	0.99	0.83	1.31
	Crossing	0.16	0.12	0.81	1.38	1.17	1.03	0.93	1.33

Risks based on accident types are shown in Table 4. From the analysis of accident data, we can know the accident type, accident vehicle, weather, time period and other information of the accident according to the location. The accident type is the main sequence, which can be divided into rear-end, reversing door switch, traffic signal violation, non-yielding, other accidents and other types. The correlation between each accident type and risk target and risk type can be sorted. Also, the cause and party of the accident under this environment can be known at the same time.

**Table 4:** The table reflects different types of accidents, main risks and main causes of accidents.

Accident type	Main risk types	Main causes of accidents
Rear-end	Car–car conflict/vehicle control	In front of the car brake/ground skid/visual blind area
Retrograde	Car–car/car–person conflict	A blind spot/sudden rush
Astern	Car–car/car–object conflict	A blind spot/sudden rush
Violation of traffic signal	Car–car/car–person conflict	The signal lamp is shielded/blind/the other party suddenly rushes out
Not yielding	Car–car/car–person conflict	Too fast

The accident types are divided into back-end, retrograde, etc., the main risk types are vehicle conflict and vehicle control, etc., and the main causes of accidents are ground anti-skid, blind spots, etc.

According to the matching of current accident location, weather and time, the priority of high-risk accident objects is increased. According to the causes of accidents in the current environment, the hidden risks are added and sorted. Therefore, the final program will output the risk target list data with priority and hidden risks.

### Target detection method based on depth learning

The models of deep learning trained are applied to identify and detect the sequence of captured images^58,61–69^. And the algorithm is used to calculate the direction speed of the target and the distance to provide data for the next step.

The velocity prediction is realized by moving the European distance of the target center point between adjacent Dayton. In short, there is a correspondence between the speeds of the real world image. If the target speed in the real world is fast, the speed in the adjacent pictures will be the same. Therefore, the speed can be obtained by finding the corresponding relation between the real speed and the video image speed. According to the shooting time of adjacent images, the frame rate, the moving distance of the target center and the moving speed in the images can be calculated. Because speed is affected by distance and time, but time is the same for real world and images, the converted distance is the most critical. What’s more, the conversion relation can be obtained by using the real size and the image size. For unmanned video images, the license plate can be selected for objects of general size. With the help of the license plates width and the actual width in the image, the conversion ratio is obtained, thus obtaining the real distance and the real speed. And the relative velocity estimation formula of the target is as follows.

Ratio of image to real world:

1 $$Scale=\frac{C}{C{^{\prime}}}$$

Actual speed size:

2 $$Speed=d\times fps\times scale$$

where *C* is the real license plate size, *C'* is the size of the object in the picture, *d* is the euclidean geometric distance of the target moving in the image determined by the displacement of the center point, and *fps* is the frame rate.

Since the velocity is vector, the velocity direction of the target should be obtained in addition to scalar. Firstly, image sequence groups within a period of time should be screened out. Secondly, the object center of the same target should be locked, and the moving direction of the object center in the sequence group should be determined to obtain the direction of instantaneous velocity.

For distance calculation, visual distortion and other issues should be considered first when CMOS sensors are used. The correction of matrix and camera internal parameters could be obtained by using Matlab camera calibration toolbox and calibration function of OpenCV library. The details will not be described here due to the length of the paper.

The system applies a fixed device to perform a single visual distance algorithm. Through conversions from real-world to camera coordinate, camera to image coordinate conversion and image to frame storage coordinate, the conversion from real world to frame storage coordinate is realized:

3 $${z}_{\nu }\left[\begin{array}{c}u\\ \nu \\ 1\end{array}\right]=\left[\begin{array}{c}{s}_{x} \, \, \, {0} \, \, \, \, {u}_{0}\\ 0 \, \, \, \, {s}_{y} \, \, \, {\nu }_{0} \, \, \\ 0 \, \, \, \, \, {0} \, \, \, \, {1}\end{array}\right]\times \left[\begin{array}{c}f \, \, \, {0} \, \, \, \, {0} \, \, \, {0}\\ 0 \, \, \, \, f \, \, \, {0} \, \, \, {0}\\ 0 \, \, \, {0} \, \, \, \, {1} \, \, \, {0}\end{array}\right]\left[\begin{array}{c}R \, \, T\\ 0 \, \, \, {1}\end{array}\right]\left[\begin{array}{c}X\\ Y\\ Z\\ 1\end{array}\right]$$

where (*X*,*Y*,*Z*) is the real world coordinate system, $({X}_{v},{Y}_{v},{Z}_{v})$ is the camera coordinate system,$({x}_{p},{y}_{p})$ is the image coordinate,$({s}_{x},{s}_{y})$ is the unit of dividing pixels by millimeters, $({u}_{0},{v}_{0})$ is the origin of the fixed frame storage coordinate system, set any position to $({\text{u}}\text{,}{\text{v}})$, *R* is the 3 × 3 rotation matrix, *T* is the 3 × 1 translation matrix, and *f* is the camera focal length. It can be simplified again:

4 $${z}_{\nu }\left[\begin{array}{c}u\\ \nu \\ 1\end{array}\right]=\left[\begin{array}{c}{f}_{x} \, \, \, {0} \, \, \, \, {u}_{0} \, \, \, {0}\\ 0 \, \, \, \, {f}_{y} \, \, \, {\nu }_{0} \, \, \, {0}\\ 0 \, \, \, \, {0} \, \, \, \, {1} \, \, \, \, \, {0}\end{array}\right]\left[\begin{array}{c}R \, \, T\\ 0 \, \, \, {1}\end{array}\right]\left[\begin{array}{c}X\\ Y\\ Z\\ 1\end{array}\right]$$

Finally reduced to:

5 $${Z}_{v}\times P={M}_{1}\cdot {M}_{2}\cdot {P}^{^{\prime}}$$

where $P$ is the frame storage coordinates, ${P}^{^{\prime}}$ is the real world coordinates, ${M}_{1}$ is the camera internal parameter matrix, ${M}_{2}$ is a camera position matrix.

Take the real situation as a profile of the Y-axis, set the *P* as the target, and project *P*y on the *Y*-axis. After deduction, the distance formula can be obtained:

6 $$d=h/\mathit{tan}\left(\mathit{arctan}\frac{Q}{h}+\mathit{arctan}\frac{H}{2f}+\mathit{arctan}\frac{{P}_{y}-{y}_{0}}{f}\right)$$

Take *Q* as the distance from the camera to the nearest point below, *h* as the camera height, *H* as the camera head height, $({x}_{0},{y}_{0})$ is the midpoint coordinate of the image. Making coordinate system conversion:

7 $$d=h/\mathit{tan}\left(\mathit{arctan}\frac{Q}{h}+\mathit{arctan}\frac{v}{2{f}_{y}}+\mathit{arc}t\mathit{an}\frac{v-{v}_{0}}{{f}_{y}}\right)$$

where $v$ is the pixel height coordinate of the target in the image, and ${v}_{0}$ and ${f}_{y}$ are internal parameters provided for calibration.

At the same time, according to its own speed calculation, some risk targets have been or will be on the collision path, and this kind of realistic risk targets are marked as the highest priority. In addition, the priority is arranged in turn according to the speed and distance of the target.

#### System construction

The prediction part first recognizes and perceives the environment, such as identifying intersections, lanes, parking lots, crosswalks, the vicinity of primary and secondary schools, etc., which is a risk model based on location and accident data. Secondly, the risk is evaluated according to historical data, that is, the risk model is used to give the prediction target and risk based on location and accident data. Finally, the target detection method based on deep learning is intended to detect the target and evaluate the risk index of the target. In a word, the system needs to solve the problems of "what is the current environment", "is there any risk in the environment", "what kind of risk is there", "the degree of danger of various risks" and "how to avoid it".

The trajectory of the risk target is predicted and tracked, and the braking distance is taken as the safe range for estimation. For hidden risks, the risk of ground skidding caused by weather will increase the braking distance, while the risk of line-of-sight problem assumes that objects with the same speed as the vehicle are located in the center of the shielding range, and estimates the safety index.

**Figure 3:**
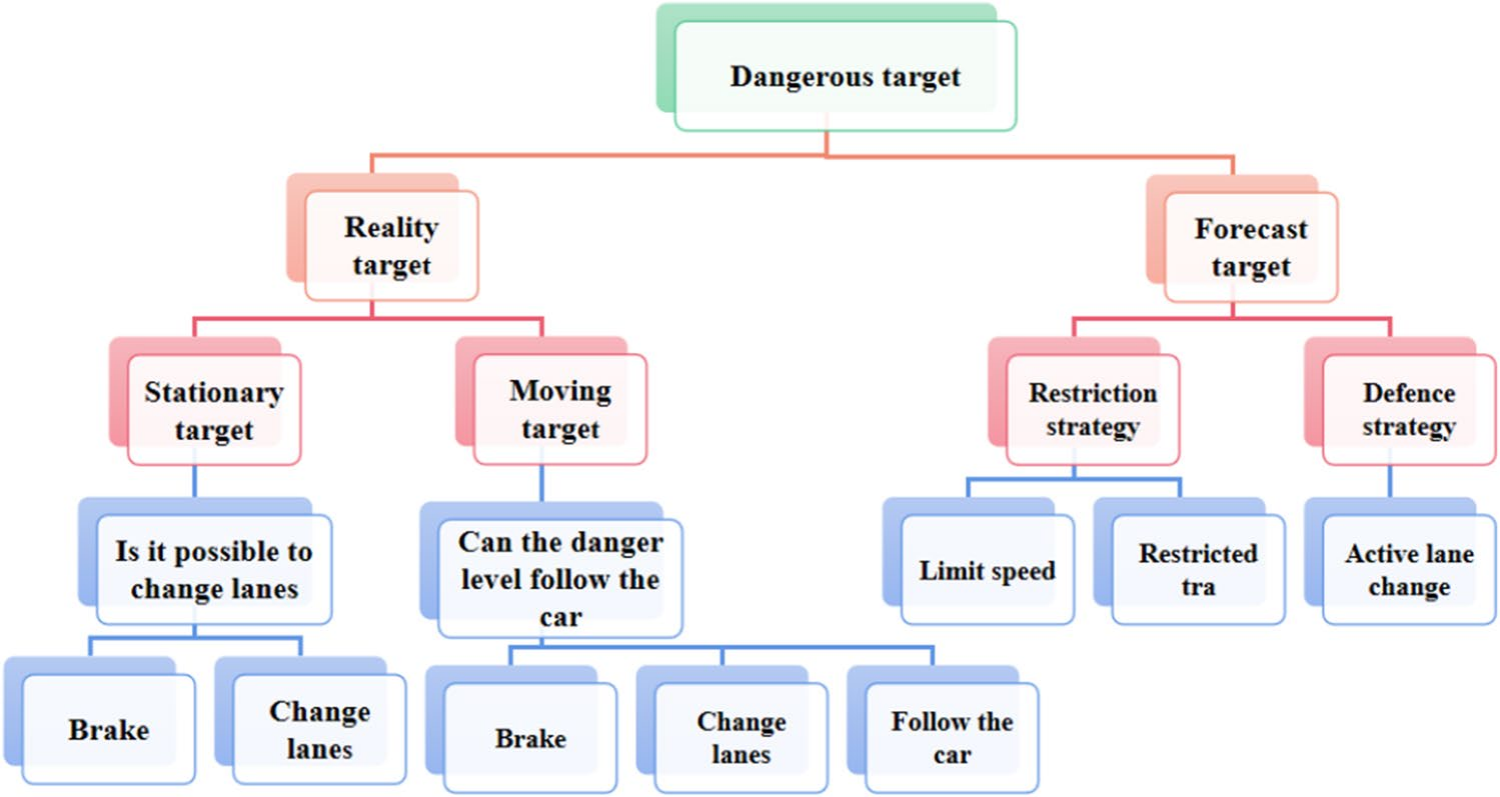
The risk model is used to give the prediction target and risk based on location and accident date, evaluate the target risk index, and then deal with it accordingly.

The parameters affecting the hazard value include the vehicle speed, braking performance, wet skid degree of the road surface and the direction of the risk target speed. Therefore, the hazard value should be obtained through comprehensive consideration of these parameters. According to relevant documents, when emergency braking is used to avoid collision, deceleration greater than 5 m/s^2^ can be considered dangerous, 2 to 5 m/s^2^ is critical danger, and below 2 m/s^2^—it can be considered safe. However, the road conditions will lead to a decrease in braking performance, which is reflected in the deceleration under the maximum braking effect, referred to as the maximum deceleration. Besides, the braking deceleration of any object should be less than the maximum, especially for objects already in the field of view. It should be considered as appropriate even for predicted objects that do not appear in the field of view. Therefore, the critical dangerous deceleration should also give priority to the environmental ground friction coefficient. Figure 3 demonstrates the internal process shown in the flow chart of circumvention algorithm.

## Construction and demonstration of algorithm model based on deep learning

### Algorithm construction of environmental perception and hazard prediction

The convolution neural network (CNN) and recursive neural network (RNN) are used to complete the task of environmental perception. The model based on LSTM variable gives different weights to different features. It can not only adapt to complex background, but also can deal with multiple targets. In addition, the end-to-end method of expression model proposed by Northwest University of science and technology in 2018 can be fully described.

Hazard prediction is divided into two parts: Target Detection and Hazard Degree prediction, in which target detection is the application scenario of deep learning. Compared with the traditional algorithm, the algorithm based on deep learning has obvious advantages in detection accuracy and efficiency. An improved SSD based target detection algorithm is proposed in this paper.

Extracting the feature information of important objects in traffic scene is the beginning of the work. Based on the supervised learning method, the attribute set is trained by multi-label classification, and the attribute prediction is carried out by training the deep convolution neural network corresponding to the loss function.

The supplementary description of environmental perception belongs to the category of image semantic recognition, and the method uesd belongs to the ‘end-to-end’.

The work of feature extraction is completed by CNN classification model. After classification, it is represented by LSTM, which is an RNN variant model. It is particularly important to note that the LSTM model is submitted not only to the extracted image features, but also to relevant information such as color, focus range of attention, location, and so on.. The feature of this method lies in dividing attention by color and weighting attention regions appropriately. However, the so-called color attention weight is to detect areas with relatively concentrated areas or large color changes of the same color in the image, especially for red and colors with significant contrast. By the way, the detection is realized by RGB color coding.

### The description of model

**Figure 4:**
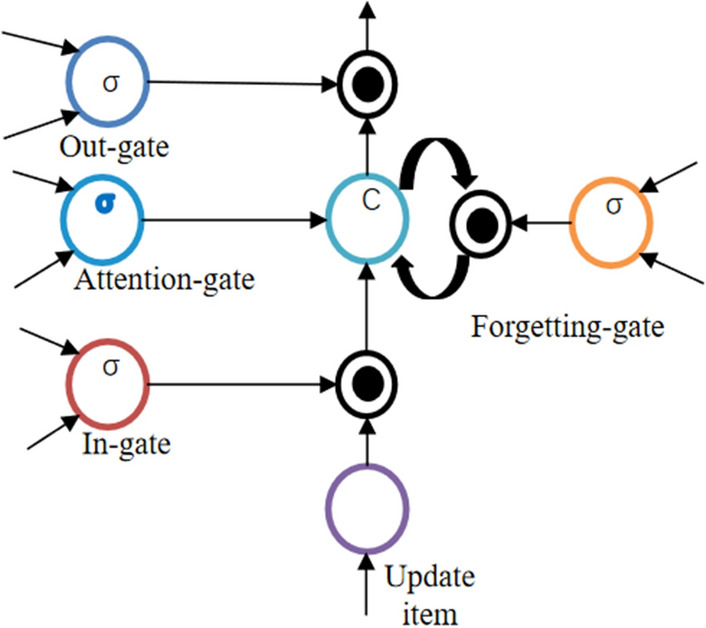
The storage unit regularly balances the content, and the replacement is controlled by four gates. Function based weight unit generation in gating phase. In addition, the hidden layer state and image features of the last node extracted by CNN are input to the unit, and the stimulation function is analyzed by machine vision.

LSTM is a special form of RNN network, whose structure has a storage unit for storing some events with certain intervals and delays in the training process. The storage unit shown in Fig. 4 regularly balances the content, and the trade-off is controlled by four gates. A feature-based weight unit is generated during the gate control phase. Besides, the hidden layer state of the previous node and the image features extracted by CNN are input to the unit, and the stimulation features are analyzed by machine vision.

During the encoding phase, pictures and labels exist as vectors in the hidden layer state. Each image extracts features with a trained VGG16 model. At the same time, the label vector is input into the LSTM model through matrix transformation. During the decoding phase, the maximum probability is obtained by multiplying the feature layer of the last layer of the hidden layer by the seventh layer of the fully connected layer. After the comparison, the output model considers the description to be the best match.

### Theoretical framework of SSD

**Figure 5:**
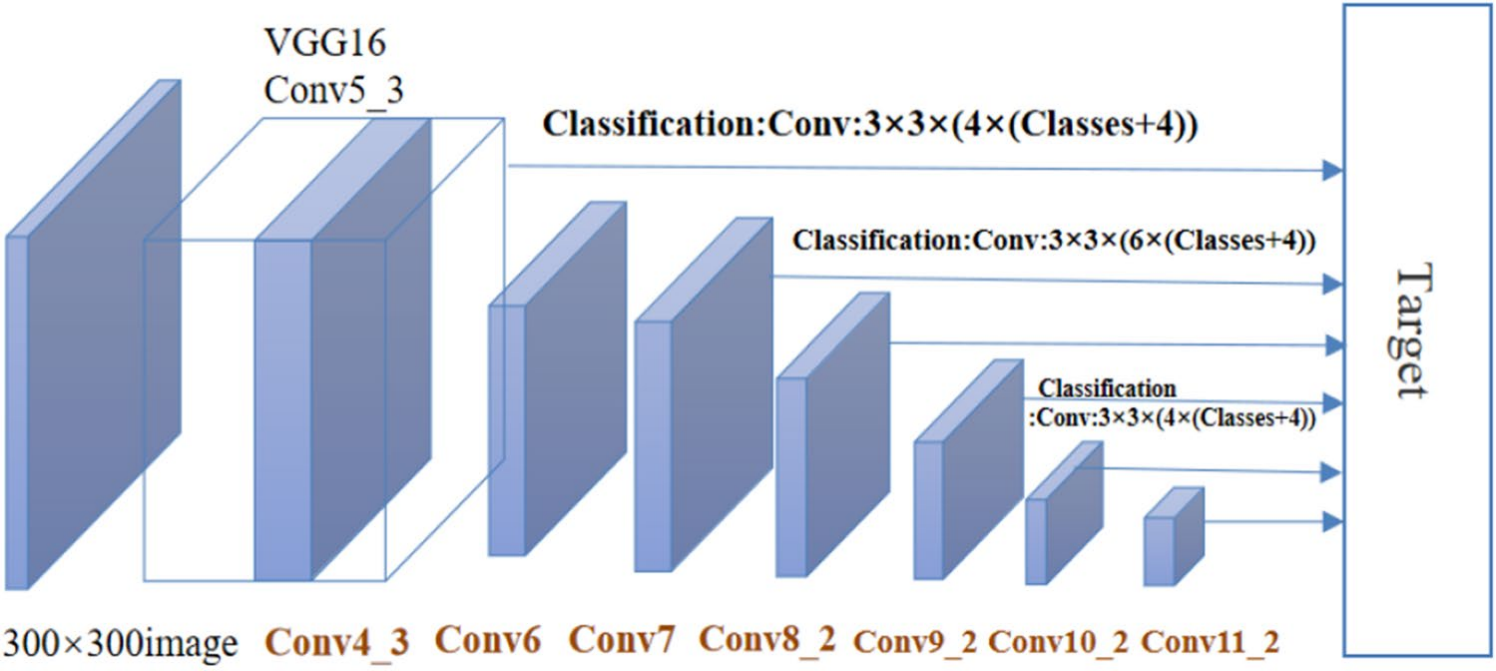
Using vgg-16 as the basic network, its extraction layer in the second half is also predicted, and detection is performed not only on other function diagrams, but also on the bottom convex 4–3 and 7 function points, so as to achieve compatibility with small goals.

In the initial SSD paper, the following structure is presented. SSD is detected using the feature pyramid structure, which uses the characteristic Feature SAMP of conv 4–3, 6–2, 7, 7–2, 8–2, 9–2. At the same time, position regression and Softmax classification are performed. Figure 5 demonstrates that SSD can use VGG-16 as the basic network. The feature extraction layer in the second half is also predicted. In addition, the detection is performed not only on the additional feature maps, but also on the underlying conv4-3 and 7-feature positions to achieve compatibility with small goals.

There are three core design concepts of SSD, as follows:

- There are two kinds of feature maps with multi-scale feature mapping: large feature mapping corresponds to small target and large target is responsible for small feature mapping.
- The feature map is extracted directly by convolution so that a large feature graph can be obtained with a relatively small convolution kernel.
- Setting a priori box each cell generates a priori box with different size, length and width. As the baseline of the bounding box, a priori frame generates multiple a priori frames in different ways during the training process.

Taking VGG16 as the basic model, the SSD transforms the fully-connected layer into 3 × 33 × 3 convolution layer CONV6 and 1 × 11 × 1 convolution layer CONV7, and pool5 from 2 × 2 to 3 × 3. Then the FC8 and drain layers are replaced by a series of convolution layers, and fine-tuned using the detection set. The Conv4 layer with a size of 38 × 38 in VGG16 will serve as the first feature map for detection. However the layer data is too large to be normalized instead.

Five feature graphs were extracted from the new layers, namely Conv7, Conv8_2, Conv9_2, Conv10_2 and Conv11_2, and six original layers of conv4 are added. Their sizes are (38, 38), (19, 19), (10, 10), (5, 5), (3, 3), (1, 1), (38, 38), (19, 19), (10, 10), (5, 5), (3, 3), (1, 1). They have different priorities, including size, length and width. What’s more, as the size of the feature map increases, the previous box size decreases.

The results are obtained by convoluting the feature graph: Category Confidence and bounding box position, each using 3 × 33 × 3 convolution is completed, the essence of SSD is dense sampling.

### Algorithm training and improvement

#### Training

##### Prior box matching

Before work, the prior frame matching with the target or part of the target is retrieved, and the matched boundary frame will enter the prediction phase. The first step of prior frame matching is to confirm Before work at least one frame to be identified. If it has a corresponding target, it becomes a positive sample, otherwise it will be a negative sample. Secondly, if there is a target matching degree greater than the threshold (generally 0.5) for the remaining negative sample, the sample will become a positive sample. Moreover, targets may have multiple prior frames that are not necessarily perfectly matched, but one prior frame cannot correspond to multiple targets.

##### Loss function

The loss function can be understood as the weighted sum of confidence and position error:

8 $$L\left(x,c,l.g\right)=\frac{1}{N}\left[{L}_{conf}\left(x,c\right)+\alpha {L}_{loc}\left(x,l,g\right)\right]$$

where *N* is the number of positive samples, ${x}_{ij}^{p}\in \left\{\mathrm{1,0}\right\}$ is used as an indication parameter, and when ${x}_{ij}^{p}=1$, the (*I-ht*) prior box matches the (*j-ht*) target with category *p*. *c* is the category confidence prediction. And *L* is the predicted value of the position, which is the position of the boundary of the target selected in the prior frame, and *g* represents its position parameter. The position error in the loss function only considers positive samples, which is defined by smooth *L*_1_ loss as follows:

9 $$L\left(x,l.g\right)={\sum }_{i\in Pos}^{N}{\sum }_{m\in \left\{cx,cy,w,h\right\}}{x}_{ij}^{k}smoot{h}_{L1}\left({l}_{i}^{m}-{\hat{g}}_{j}^{m}\right)$$

10 $${\widehat{g}}_{j}^{cx}=\frac{\left({g}_{j}^{cx}-{d}_{i}^{cx}\right)}{{d}_{i}^{w}}$$

11 $${\widehat{g}}_{j}^{cy}=\frac{\left({g}_{j}^{cy}-{d}_{i}^{cy}\right)}{{d}_{i}^{h}}$$

12 $${\widehat{g}}_{j}^{w}=log\frac{\left({g}_{j}^{w}\right)}{{d}_{i}^{w}}$$

13 $${\widehat{g}}_{j}^{h}=log\frac{\left({g}_{j}^{h}\right)}{{d}_{i}^{h}}$$

14 $${smooth}_{{L}_{1}}(x)=\left\{\begin{array}{cc}0.5{x}^{2}& \, \text{if |x|<}{1}\\ |x|-0.5& \, {\text{otherwise}}\end{array}\right.$$

The parameters are as follows:

15 $${\widehat{g}}_{j}^{cx}=\frac{\frac{\left({g}_{j}^{cx}-{d}_{i}^{cx}\right)}{{d}_{i}^{w}}}{\text{variance}}$$

16 $${\widehat{g}}_{j}^{cy}=\frac{\frac{\left({g}_{j}^{cy}-{d}_{i}^{cy}\right)}{{d}_{i}^{h}}}{\text{variance}}$$

17 $${\widehat{g}}_{j}^{w}=\frac{\mathit{log}\left(\frac{{g}_{j}^{w}}{{d}_{i}^{w}}\right)}{\text{variance}}$$

18 $${\widehat{g}}_{j}^{h}=\frac{\mathit{log}\left(\frac{{g}_{j}^{h}}{{d}_{i}^{h}}\right)}{\text{variance}}$$

For confidence error, it adopts softmax loss:

19 $${L}_{conf}\left(x,c\right)=-{\sum }_{i\in Pos}^{N}{x}_{ij}^{p}\mathit{log}\left(\frac{\mathit{exp}\left({c}_{i}^{p}\right)}{\sum_{p}exp({c}_{i}^{p})}\right)-\sum_{i\in Neg}log({\widehat{c}}_{i}^{0})$$

#### Improvement based on focal loss

**Figure 6:**
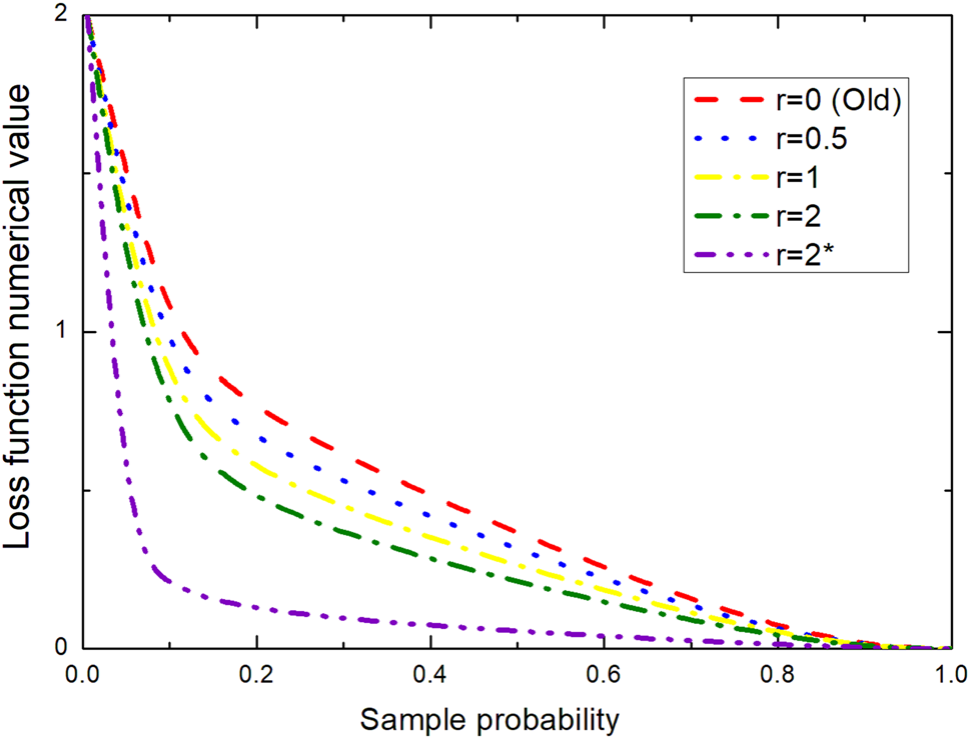
This figure fits the linear relationship between loss function and sample probability when r = 0, r = 0.5, r = 1, etc. Through it, we can clearly see the relationship between lost function numerical value and sample probability.

The main reason why single-level detection is not as accurate as two-level detection is the imbalance of sample categories. Category imbalance will bring too many negative samples, which account for most of the loss function. Therefore, the focal loss is proposed as a new loss function. The loss function is modified According to the standard cross entropy loss in Fig. 6. This function can reduce the samples that are easy to classify by changing the evaluation method, so as to apply more weights to the samples that are difficult to classify in the training process. The formula is as follows:

20 $$FL\left({p}_{t}\right)=-\alpha t{\left(1-{p}_{t}\right)}^{\Upsilon}\mathit{log}\left({p}_{t}\right)$$

Firstly, a factor is added to the original standard cross entropy loss, thereby reducing the loss of easily classified samples. This makes us pay more attention to difficult and misclassified samples. For example, *γ* = 2, for a positive sample with a prediction result of 0.95, value of the loss function becomes smaller because the power of (1–0.95) is very small. However, for negative samples with a prediction probability of 0.3, the loss becomes relatively large, which is achieved by suppressing the loss of positive samples.

Therefore, the new method pays more attention to this indistinguishable sample. In this way, the influence of simple samples is reduced, and the effect will be more effective only when a large number of samples with low prediction probability are added together. Meanwhile more penalties are required for easily distinguishable negative samples. The actual formula is as follows:

21 $$L_{{fl}} = \left\{ {\begin{array}{*{20}l} { - \alpha {\text{ (}}1 - y^{\prime } )^{\gamma } log\;y^{\prime } } \hfill & {y = {\text{postive}}\;{\text{simple}}} \hfill \\ { - (1 - \alpha )y^{{\prime \gamma }} {\text{ }}log({\text{1}} - y^{\prime } {\text{),}}} \hfill & {{\text{y}} = {\text{negtive}}\;{\text{simple}}} \hfill \\ \end{array} } \right.$$

In the experiment, *γ* = 2 and *α* = 0.25 have the best effect.

#### Improvement based on KL loss

The traditional boundary box regression loss (i.e., Smooth *L*1 loss) does not take the deviation of the actual boundary on the ground into consideration. When the classification score is very high, the regression function is considered to be accurate, but it is not always the case.

Bounding box prediction is modeled as Gaussian distribution, and the boundary box of positive samples is modeled as a Dirac delta function. The asymmetry of these two distributions is measured by KL divergence. When KL divergence approaches 0, these two distributions are very similar. KL loss is the KL divergence of minimizing the Gaussian distribution predicted by the bounding box and the Dirac delta distribution of positive samples. In other words, KL loss makes the bounding box prediction approximate gaussian distribution and close to positive samples. And it converts the confidence into the standard deviation of the bounding box prediction.

The two probability distributions *P* and *Q* of a discrete or continuous random variable whose *KL* divergence is defined as:

22 $$D(P\| Q)={\sum }_{i\in X}P(i)*\left[\mathit{log}\left(\frac{P(i)}{Q(i)}\right)\right]$$

23 $$D(P\| Q)={\int }_{x}P(x)*\left[\mathit{log}\left(\frac{P(i)}{Q(i)}\right)\right]dx$$

Before calculating *KL* divergence, the bounding box needs to be parameterized.$\left({x}_{1},{y}_{1},{x}_{2},{y}_{2}\right)$ is the upper left and lower right coordinates of the prediction bounding box.$\left({x}_{1}^{*},{y}_{1}^{*},{x}_{2}^{*},{y}_{2}^{*}\right)$ is the coordinates of the upper left and lower right corners of the real box.$\left({x}_{1a},{y}_{1a},{x}_{2a},{y}_{2a},{h}_{a},{w}_{a}\right)$ is an anchored bounding box generated by aggregating all real boxes. Then the deviations of the predicted and real bounding boxes are as follows:

24 $${t}_{x1}=\frac{{x}_{1}-{x}_{1a}}{{w}_{a}},{t}_{x2}=\frac{{x}_{2}-{x}_{2a}}{{w}_{a}}$$

25 $${t}_{y1}=\frac{{y}_{1}-{y}_{1a}}{{h}_{a}},{t}_{y2}=\frac{{y}_{2}-{y}_{2a}}{{h}_{a}}$$

26 $${t}_{x1}^{*}=\frac{{x}_{1}^{*}-{x}_{1a}}{{w}_{a}},{t}_{x2}^{*}=\frac{{x}_{2}^{*}-{x}_{2a}}{{w}_{a}}$$

27 $${t}_{y1}^{*}=\frac{{y}_{1}^{*}-{y}_{1a}}{{h}_{a}},{t}_{y2}^{*}=\frac{{y}_{2}^{*}-{y}_{2a}}{{h}_{a}}$$

Similarly, the parameter without * indicates the deviation between the prediction and the anchored boundary frame, and the parameter with * indicates the deviation between the real and the anchored boundary frame.

Assuming that the coordinates are independent, a univariate Gaussian function is used for simplicity:

28 $${P}_{\Theta }(x)=\frac{1}{\sqrt{2\pi {\sigma }^{2}}}{e}^{-\frac{{\left(x-{x}_{e}\right)}^{2}}{2{\sigma }^{2}}}$$

where *x*_*e*_ is the estimated boundary box position and the standard deviation *σ* is the estimated uncertainty. When *σ* → 0, the position accuracy of boundary box is very high.

The real boundary box on the ground can also be expressed by Gaussian distribution, and becomes Dirac delta function when *σ* → 0:

29 $${P}_{D}(x)=\delta \left(x-{x}_{g}\right)$$

where *x*_*g*_ is the real boundary box position on the ground. At this point, we can construct a bounding box regression function with *KL* loss, and establish a formula to minimize the *KL* error of *P *_*θ*_ (*x*) and *P*_*D*_ (*x*) on *N* samples:

30 $$\widehat{\Theta }=\underset{\Theta }{argmin}\frac{1}{N}\sum {D}_{KL}\left({P}_{D}(x)\| {P}_{\Theta }(x)\right)$$

*KL* divergence is used as the loss function *L*_*reg*_ for bounding box regression, and the classification loss *L*_*cls*_ remains unchanged. For a single sample:

31 $$\begin{aligned} L_{reg} & = D_{KL} \left( {P_{D} (x)||P_{\Theta } (x)} \right) \\ & = \smallint P_{D} (x)\log P_{D} (x)dx - \smallint P_{D} (x)\log P_{\Theta } (x)dx \\ & = \frac{{\left( {x_{g} - x_{e} } \right)^{2} }}{{2\sigma^{2} }} + \frac{{\log (\sigma^{2} )}}{2} + \frac{\log (2\pi )}{2} - H(P_{D} (x)) \\ \end{aligned}$$

When the prediction of the bounding box is inaccurate, because the prediction closer to the real bounding box is certainly stable and its variance is small, the smallest possible variance can reduce *L*_*reg*_. After the variance of the predicted position of the bounding box is obtained, the candidate positions are voted according to the known variance of adjacent bounding boxes. Besides, the candidate coordinate values with the largest score are selected to be weighted to update the coordinates of the bounding box, so as to make the positioning more accurate. What’s more, border boxes with lower positions and lower colors have higher weights. The new coordinates are calculated as follows:

32 $$\begin{array}{cc}\begin{array}{c}{p}_{i}={e}^{-{\left(1-IoU\left({b}_{i},b\right)\right)}^{2}/{\sigma }_{t}}\\ x=\frac{{\sum }_{i}{p}_{i}{x}_{i}/{\sigma }_{x,i}^{2}}{{\sum }_{i}{p}_{i}/{\sigma }_{x,i}^{2}} \end{array}& \text{subject to }IoU\left({b}_{i},b\right)>0\end{array}$$

where ${\sigma }_{t}$ is an adjustable parameter for variable voting. When $IoU\left({b}_{i},b\right)$ is larger, ${p}_{i}$ is larger, the two bounding boxes overlap each other more and do the same for the remaining coordinate values. SSD detects the generated preselected box computing loss through *FL* loss function classification and boundary regression. Besides, the boundary regression of SSD is improved based on *KL* loss method. Frames with large variance and adjacent boundary frames containing the selected frames but too small will get low scores when voting. Moreover, the SSD algorithm can effectively avoid the above anomalies by variance voting instead of *IoU* overlap degree.

### Model testing and analysis

The environment perception is divided into two parts, the micro part is the main perception of the scene by machine vision, which is used to confirm and supplement the macro and micro perception.

First of all, we tested the *Roi* weighting using live campus photos taken on May 7, 2020. The advantage of this algorithm is that the region of interest can be identified first, and then the further perception can be completed. Therefore, the region of interest test was performed first, and the effect of attention weighting was significant.

Second, the environment perception test was carried out because the region of interest was weighted and the weighted region was described firstly. After testing, the algorithm can complete the perception of the simple traffic scene and recognize the red light of the intersection, the bus and the right-turn sign on the road, and can supplement and confirm the environment perception part.

At the same time, different databases, Google Nic, Log BILINEAR and other different algorithms are used to compare with experiments, because the Algorithm has good performance on Flickr8K, Flickr30K and MS COCO databases, and validated the experimental results of the Northwestern Polytechnic University team. The experimental results on the Flickr8K database are shown in Table 5, Flickr30K database are shown in Table 6, and MS COCO database are shown in Table 7.

**Table 5:** The results of the experiments on the database Flickr8K.

Ways	BLEU-1	BLEU-2	BLEU-3	BLEU-4
Google NIC	63	41	27	–
Log bilinear	65.6	42.4	27.7	17.7
The design algorithm	67.2	45.8	32.1	18.9

Methods are divided into Google NIC, Log Bilinear and The design algorithm.

**Table 6:** The results of the experiments on the database Flickr30K.

Ways	BLEU-2	BLEU-3	BLEU-4
Google NIC	42.3	27.7	18.3
Log bilinear	38	25.4	17.1
The design algorithm	45.1	29.9	21.1

Methods are divided into Google NIC, Log Bilinear and The design algorithm.

**Table 7:** The results of the experiments on the database MS COCO.

Ways	BLEU-1	BLEU-2	BLEU-3	BLEU-4
Google NIC	66.6	46.1	32.9	24.6
Log Bilinear	70.8	48.9	34.4	24.3
The design algorithm	72.3	51.8	37.1	25.1

Methods are divided into Google NIC, Log Bilinear and The design algorithm.

**Figure 7:**
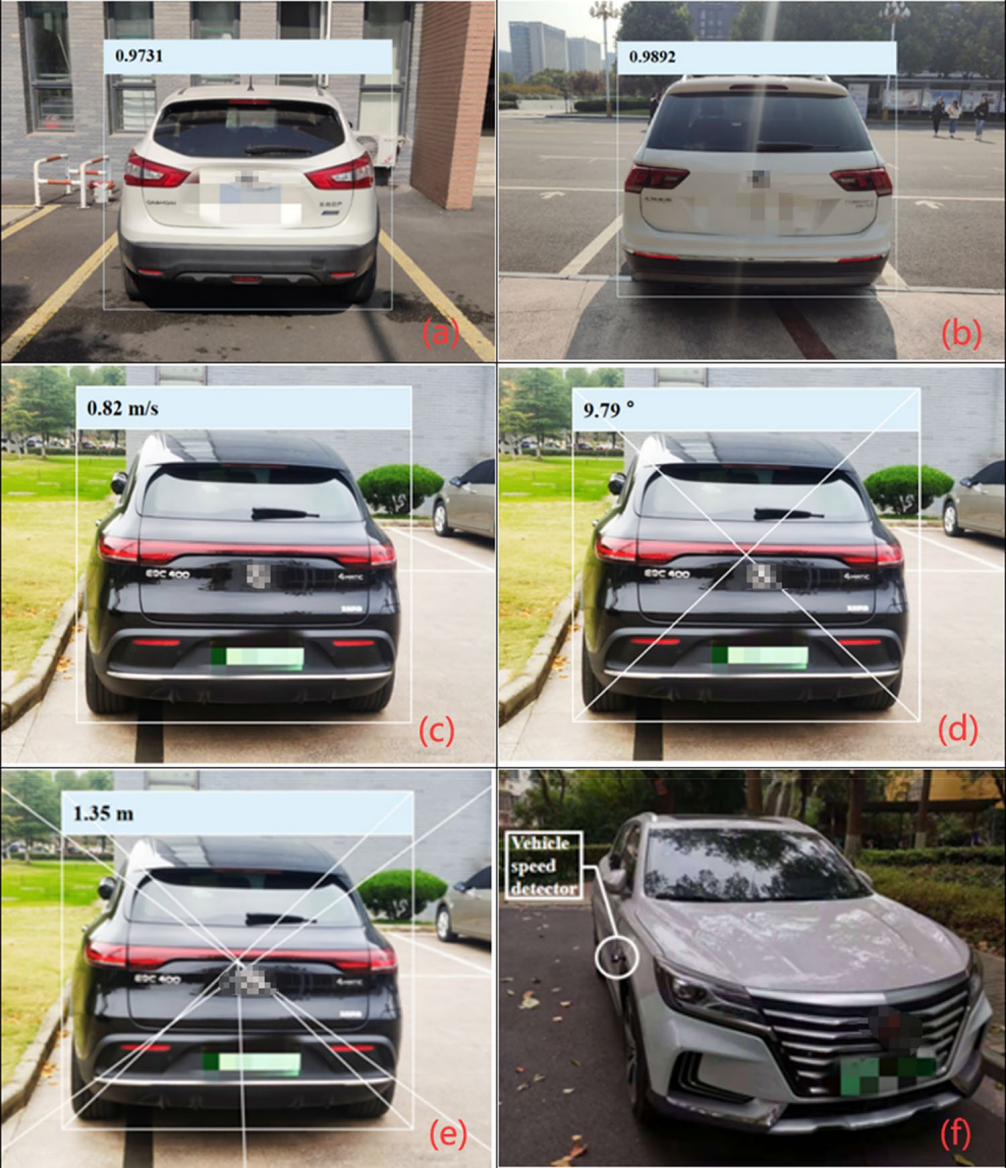
(**a**, **b**) Detect the dynamic vehicle, including its speed and driving direction. As shown in the figure, the distance between the vehicle and the parking space line is 0.9731 m and 0.9892 m respectively. (**c**, **d)** Dynamic vehicle detection mainly detects its driving speed and direction. As shown in the figure, the vehicle speed is 0.82 m/s and the driving direction is 9.79° to the left. (**e**, **f**) The figure shows that the vehicle speed detector is used to detect the actual vehicle and can accurately detect its driving speed and direction.

The focus will be on target detection in the hazard prediction section. First of all, the vehicle test is carried out by using field test maps and data set pictures. Secondly, dynamic vehicles need to be detected, including their speed, distance and running direction. The vehicle target detection is shown in Fig. 7a and b. The dynamic vehicle direction estimation is shown in Fig. 7c and d. The dynamic vehicle distance estimation is shown in Fig. 7e. The vehicle speed detector is used to detect the speed of the dynamic vehicle in Fig. 7f.

## Results and discussion

### Simulated route

**Figure 8:**
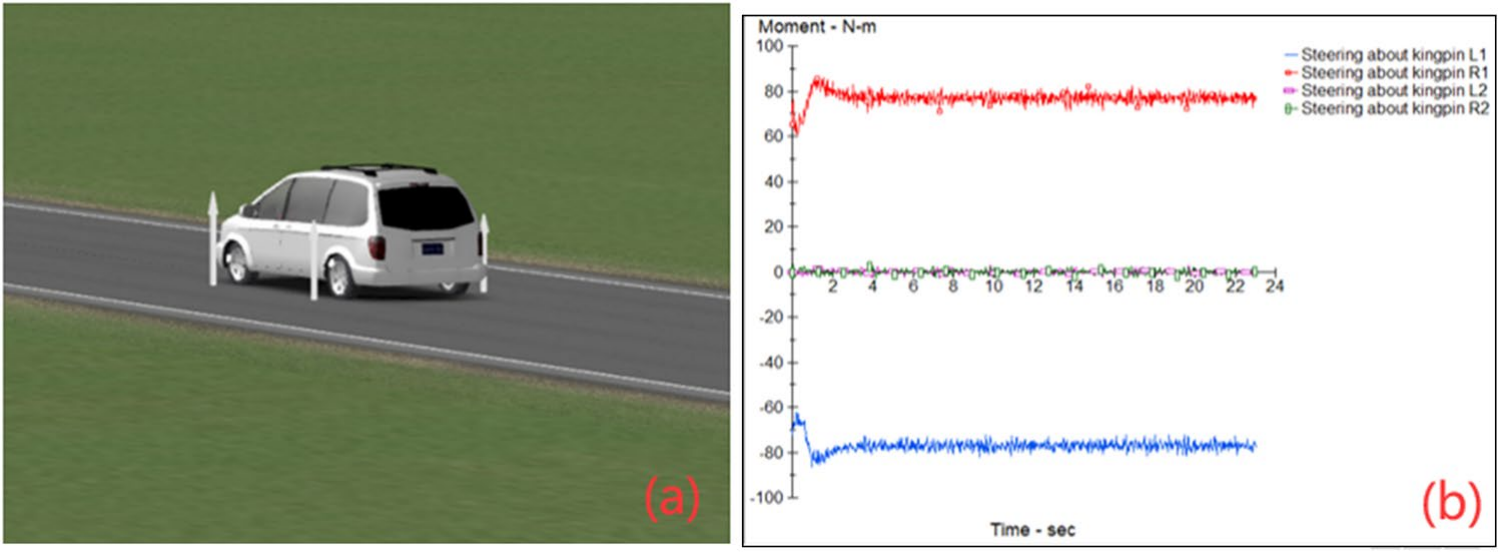
Simulation interface of CarSim. (**a**) Simulate vehicle operation by adjusting scene, road surface and definition, driving conditions, etc. (**b**) It shows the speed constraints of the simulation system output in the whole simulation driving process.

In order to better reflect the function of the system, the paper uses Matlab and CarSim to set up the dangerous situation of vehicle crossing under different conditions and conduct a joint simulation. The simulation system will output the speed constraint throughout the whole simulated driving process. Figure 8a simulates the vehicle operation by adjusting the scene, road surface and definition, driving conditions, etc. Figure 8b shows the speed constraint of the simulation system output in the whole simulation driving process.

Choose the route from school to the bus station. The path passes through two campuses, two residential areas, a commercial center and four intersections. The total length of this path is 5.6 km, which can meet the needs of system test and simulation. In order to facilitate the simulation, the path of latitude and longitude are sampled. In addition, the path can be divided into two paths, Tianshan Road and Youth road, and the results are shown in Tables 8 and 9 respectively.

**Table 8:** The table mainly includes location, longitude and latitude coordinates and remarks.

Positions	Latitude and longitude coordinates	Remarks
Starting point	120.205976, 33.37899	West Gate of Yancheng Normal University's New Long Campus
Tianshan road	120.20524, 33.37991	–
Crossroads	120.204431, 33.380875	Century avenue and Tianshan Road Intersect
Tianshan road	120.202715, 33.382887	–
Yancheng institute of technology	120.201997, 33.38361	–
Tianshan road	120.200865, 33.384786	–
Tianshan road	120.198403, 33.387544	Unknown path junction
Xinfeng community	120.198403, 33.387544	–
Crossroads	120.195753, 33.390318	Tianshan Road and Youth Road Intersect

The location is divided into starting point and Tianshan Road. The longitude and latitude coordinates are consistent with the location.

**Table 9:** The table mainly includes location, longitude and latitude coordinates and remarks.

Positions	Latitude and longitude coordinates	Remarks
Youth road	120.193714, 33.389948	–
Tongyu River Bridge	120.190229, 33.388803	–
Tongyu River Bridge	120.186968, 33.38768	There is a side road junction
Youth road	120.181596, 33.385909	There is a side road junction
Complex intersection	120.180096, 33.385344	Youth Road and Fangong Road Intersect
Howard square	120.174985, 33.382133	–
Crossroads	120.173907, 33.381576	Youth Road and Wengang Road Intersect
Youth road	120.176206, 33.382812	–
End	120.178425, 33.384033	Tianshan Road and Youth Road Intersect

The location is divided into Youth road and Tongyu River Bridge. The longitude and latitude coordinates are consistent with the location.

Latitude and longitude sampling table of Tianshan Road is shown in Table 10. Longitude and latitude sampling table of Youth Road is shown in Table 11. Based on the collected coordinates of latitude and longitude, the whole system can be simulated and tested. In Matlab, the REGEXP function can be used to get a Web page, so as to get the location name directly through the map API and the output environment data through Json. Then the starting latitude and longitude for the test are selected to successfully obtain the remote data.

**Table 10:** The table mainly includes location, longitude and latitude coordinates and remarks.

Positions	Latitude and longitude coordinates	Remarks
Starting point	120.205976, 33.37899	West Gate of Yancheng Normal University's New Long Campus
Tianshan road	120.20524, 33.37991	–
Crossroads	120.204431, 33.380875	Century avenue and Tianshan Road Intersect
Tianshan road	120.202715, 33.382887	–
Yancheng institute of technology	120.201997, 33.38361	–
Tianshan road	120.200865, 33.384786	–
Tianshan road	120.198403, 33.387544	Unknown path junction
Xinfeng community	120.198403, 33.387544	–
Crossroads	120.195753, 33.390318	Tianshan Road and Youth Road Intersect

The location is divided into Starting point and Tianshan road. The longitude and latitude coordinates are consistent with the location.

**Table 11:** The table mainly includes location, longitude and latitude coordinates and remarks.

Positions	Latitude and longitude coordinates	Remarks
Youth road	120.193714, 33.389948	–
Tongyu River Bridge	120.190229, 33.388803	–
Tongyu River Bridge	120.186968, 33.38768	There is a side road junction
Youth road	120.181596, 33.385909	There is a side road junction
Complex intersection	120.180096, 33.385344	Youth Road and Fangong Road Intersect
Howard square	120.174985, 33.382133	–
Crossroads	120.173907, 33.381576	Youth Road and Wengang Road Intersect
Youth road	120.176206, 33.382812	–
End	120.178425, 33.384033	Tianshan Road and Youth Road Intersect

The location is divided into Youth road and Tongyu River Bridge. The longitude and latitude coordinates are consistent with the location.

### System simulation

In order to facilitate the simulation of the system function, the speed constraint on the simulation path is visualized. Considering the unity of safety and efficiency, the time has a great influence on speed constraint. Assuming that the vehicle is traveling at 60 km/h, simulation speed constraints are provided on Monday, Sunday at 8:00 and Monday at 8:00 and 23:00. By the way, the system will adjust appropriately according to the risk weighting.

The speed constraint was loaded into CarSim for dynamic simulation, and the data at 23:00 on Monday was selected to check the difference between simulations with and without the system.

What’s more, the system will adjust appropriately according to the risk weighting. The speed constraint was loaded into CarSim for dynamic simulation, and the data at 23:00 on Monday was selected to check the difference between simulations with and without the system.

**Figure 9:**
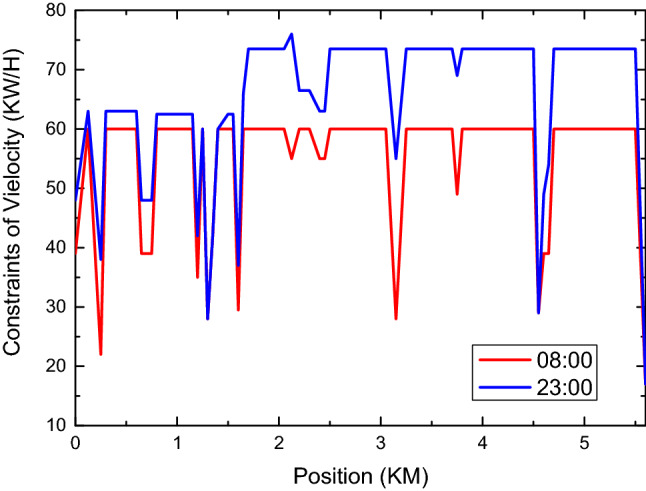
This figure reflects the constrains of velocity between 8 a.m. and 11 p.m. From the figure, we can see that the value of 11 p.m. is greater than that of 8 a.m.

The comparison of speed constraints between Monday and Sunday is shown in Fig. 9. On the whole, the speed constraints on Monday are stricter than those on Sunday, which is caused by risk weighting based on experience. And on the basis of time weighting, roads and intersections of different levels are weighted simultaneously by the system, and corresponding speed constraints are finally formed. At this time, the speed constraint does not consider the vehicle dynamics or the comfort of driving. In practical application, the speed constraint needs to consider the acceleration required of the current speed of the vehicle to implement the speed constraint. The acceleration needs to be comprehensively considered according to the center of gravity, braking performance, acceleration performance, ground friction coefficient, etc., which are ignored during the simulation.

**Figure 10:**
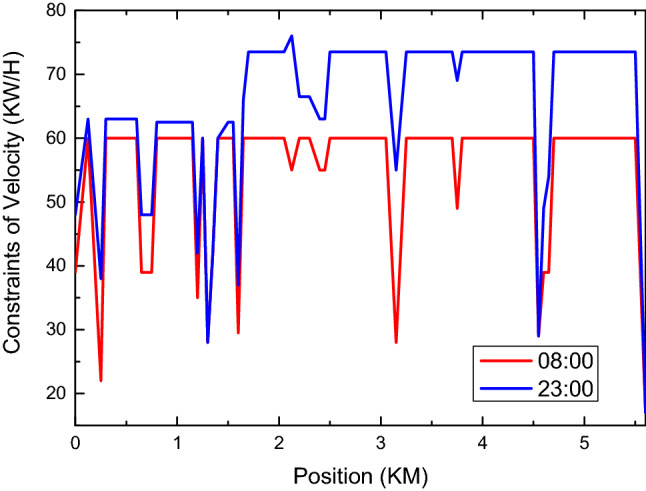
This figure reflects the change law of speed between 8 a.m. and 11 p.m. it can be seen from this figure that the speed limit at 11 p.m. is significantly greater than that at 8 a.m.

The comparison results of speed constraints at different times on the same day are shown in Fig. 10. The speed constraint at 23:00 has been relaxed and vehicles are allowed to travel beyond the standard speed. In practical application, the unmanned driving system needs to combine the road supervision situation with the traffic situation on site to execute the speed. This speed only outputs speed constraints from the perspective of environmental hazards and does not represent the final execution speed.

**Figure 11:**
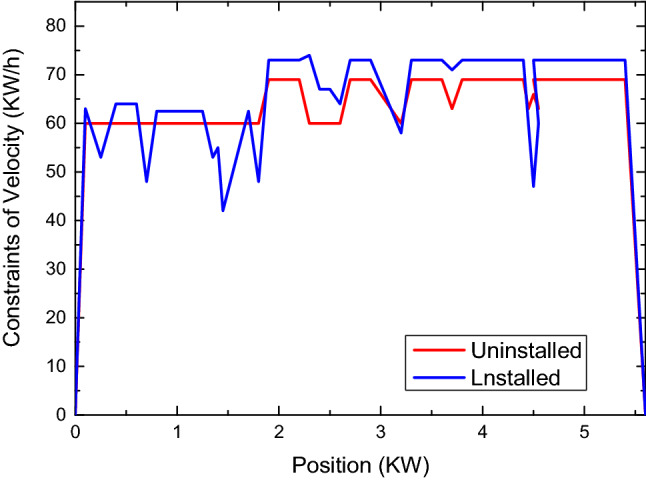
This figure reflects the change law of speed limit between unloading and installation points. It can be seen from this figure that the change of unloading speed limit is stable and the speed limit fluctuates greatly during installation.

The comparison of simulated driving speeds between vehicles equipped with the system and human-driven vehicles is shown in Fig. 11. Since most of the front part of the simulated route passes schools and intersections while other parts are expressways with few intersections, different driving speeds are simulated on the basis of actual driving. Under the ideal condition of smooth traffic, human driving vehicles will be affected by road grade, traffic control and subjective judgment. Besides, the driving speed of vehicles equipped with this system is similar to that of human beings in trend, and the speed constraint is strictly implemented according to the risk grade. It can be seen that the vehicles can realize the defensive driving of human beings more intelligently and flexibly, relying on accurate scientific and objective data analysis conclusions instead of subjective experience.

In order to reflect the efficiency of the system more particularly, the application test of the vehicle with or without the system is carried out through the car accident simulation built in CarSim.

The content of the traffic accident simulation is that an oncoming vehicle with a speed exceeding 100 km/h strays into the lane while avoiding the normally running vehicle and eventually rolls over. The normally running vehicle with a speed of 100 km/h completes emergency braking during the avoidance. The accident site is a freeway.

In the CarSim simulation, the speed of 70 km/h is set as the normal driving speed, which is consistent with the actual use of expressway. Within a period of visible sight distance, vehicles were not observed to enter the lane in opposite direction. The collision was avoided by emergency braking. The whole braking process has a great influence on passengers and uncertainty factors. The danger has been successfully avoided. The simulation results indicate the effectiveness of the system.

## Conclusion

Environmental hazard prediction and avoidance technology is the key in the research field of unmanned vehicles, which provides an important guarantee for the driving of unmanned vehicles in the real environment. Nowadays, most unmanned driving systems are equipped with hazard prediction and avoidance systems. However, environment-oriented data-based environmental hazard prediction and avoidance technology has not been developed enough. In this paper, Matlab and CarSim are used to simulate the entire system. The speed constraints and simulation speed diagrams under various condition are output on the selected drive path to verify the effectiveness of the system function. The system is innovative to solve the problems of unmanned environmental hazard in the target detection. The next work is to experiment with hyper-parameter tuning and model training by real-world observations in the further research.

## List of symbols

*C*: Real license plate size
*d*: Euclidean geometric distance
*P*: Frame storage coordinates
*M*_*1*_: Camera internal parameter matrix
*H*: Camera head height
*h*: Camera height
*ν*_*0*_,*f*_*y*_: Internal parameters provided for calibration
*σ*: Estimated uncertainty
*x*_*e*_: Estimated boundary box position and standard deviation
*σ*_*t*_: Adjustable parameter for variable voting
*P*_*y*_: Target of P on the Y-axis
*L*_*reg*_: Bounding box regression
*t*_*xi*_*, t*_*yi*_: Deviation between the prediction and the anchor boundary frame
*x*_*i*_, *y*_*i*_: Bounding box position in the axle
*C′*: Size of the object in the picture
*fps*: Frame rate
*P’*: Frame storage coordinates
*M*_2_: Camera position matrix
*f*: Camera focal length
*g*: Position parameter
*ν*: Pixel height coordinate of the target in the image
*c*: Category confidence prediction
*x*_*g*_: Real boundary box position on the ground
*Q*: Istance from the camera to the nearest point below
*D*: Kullback–Leibler divergence
*L*_*cls*_: Classification loss
*t*_*xi*_^***^*, t*_*yi*_^***^: Deviation between the real and the anchor boundary frame
*x*_*i*_^***^, *y*_*i*_^***^: True box position in the axle
